# Supplementary material for: Novel polymer fixed-target microfluidic platforms with an ultra-thin moisture barrier for serial macromolecular crystallography
Source: bioRxiv. 2025 Jul 18:2025.07.13.663488. Preprint. [Version 1] doi: 10.1101/2025.07.13.663488 (PMC12338584; doi:10.1101/2025.07.13.663488)
Supplement: Supplement 1 — Fig. S1: a) Large area free-standing PaN film. These films are cut to size to make different types of PaN fixed-target sample platforms; b) Closed-chip PaN FT with independent windows; c) REP-24 microcrystals sample loading on film-on-film PaN FT and held in place to the actuators using SOS metal holder49; d) Film-on-film PaN FT loaded with REP-24 wild type protein microcrystals. Fig. S2. Systematic Screen printing methodology of making closed-chip PaN FT. a) The PaN film is cut to size, and placed on the screen-coating instrument, and held in place with vacuum; b) A stell-mesh with the desired pattern is placed on the film, and a small quantity of PDMS resin is introduced and the squeegee is applied; c) The resulting pattern with the PaN film is baked at 100°C; d) Two patterned films are plasma treated; e) The plasma treated films are placed one-upon-another and bonded; f) The ready to use assembled devices have holes at the desired locations for crystal slurry introduction. Fig S3. Automated vacuum-holder based screen printer utilized for fabricating closed chip PaN FT. [file media-1.pdf]

LLNL-JRNL-2007768

Novel polymer fixed-target microfluidic platforms with an ultra-thin moisture barrier for serial macromolecular crystallography

Sankar Raju Narayanasamy [1], Megan L. Shelby [1], Chandraki Chatterjee [1], Jenny Zhou[2], Samuel Rose[3], Julian Orlans [3], Swagatha Ghosh[4], Anne Marie Cardenas[2], Sabine Botha[5], Kevin Gu[6], Donald Petit[7], Zhongrui Liu [6], Francesco Fornasiero [1], Stella Lisova[8], Elyse Schriber [8], Daniel Rosenberg [8], Thej Tumkur Umanath[9], Silvia Russi[10], Brent Segelke [1], Tonya L. Kuhl [6], Martin Trebbin [7,11], Shibom Basu [12], Daniele de Sanctis [3], Matthias Frank[1,13]*

*Corresponding author (frank1@llnl.gov)

1. Biosciences and Biotechnology Division, Physical and Life Sciences Directorate, Lawrence Livermore National Laboratory, Livermore, USA; 2. Materials Engineering Division, Engineering Directorate, Lawrence Livermore National Laboratory, Livermore, USA; 3. ESRF- The European Synchrotron, Grenoble, France; 4. Department of Applied Physics, Graduate School of Engineering, Nagoya University; Nagoya, Japan; 5. Department of Physics, Arizona State University, Arizona, USA; 6. Department of Chemical Engineering, University of California, Davis, USA; 7. Department of Chemistry, The State University of New York at Buffalo, Amherst, USA; 8. Linac Coherent Light Source, SLAC National Accelerator Laboratory, Menlo Park, USA; 9. Materials Science Division, Physical and Life Sciences Directorate, Lawrence Livermore National Laboratory, Livermore, USA; 10. Stanford Synchrotron Radiation Light Source, SLAC National Accelerator Laboratory, Menlo Park, USA; 11. Hauptman-Woodward Medical Research Institute, Buffalo, USA; 12. European Molecular Biology Laboratory, Grenoble, France; 13. Department of Biochemistry and Molecular Medicine, School of Medicine, University of California, Davis, USA.

Supplementary Material

Parylene coater handling procedure:

The following procedure is followed to obtain nearly uniform Parylene-N deposition on the 6” wafer. SCS parylene coater is utilized to fabricate the free-standing PaN films.

1. Inspect and clean the Cold Trap

- Check for parylene build-up.
- If build-up is present, clean the cold trap thoroughly.
- After cleaning, coat all surfaces with diluted Micro-90 to prevent adhesion of deposited material.

2. Prepare the Chamber

- Remove the chamber lid and place it upside down on a clean surface. Ensure the O-ring does not touch any surface.
- Using Scotch tape, remove debris from the chamber lid’s O-ring. Confirm the O-ring is clean and intact.
- Inspect the port holes inside the chamber and ensure they are not obstructed by parylene.

3. Load silicon wafers

- Place the silicon wafers in the center of the chamber.
-

4. Reassemble the Chamber

- Confirm that the O-ring seating area is free of debris.
- Carefully reposition the chamber lid, ensuring a flush seal with no visible gaps.

5. Load Parylene Dimer

- Prepare a boat-shaped aluminum foil container.
- Weigh out the desired amount of dimer and place it into the boat.
- Open both the dimer chamber and the secondary chamber.
    - Inspect and clean O-rings as necessary.
- Load the dimer-containing boat into the chamber.

- Close all chamber doors securely.

6. Install the Cold Trap

- Ensure the O-ring on the cold trap is clean before placing it into position.

7. Power On System

- If previously pressed, depress the EMO button.
- Switch on main power and allow the system to reset.

8. Confirm Deposition Parameters

- Input the appropriate deposition parameters for the experiment.

9. Initiate Vacuum

- Turn on the vacuum system and allow to run for 15 minutes.

10. Cool the Cold Trap

- Switch on the chiller and wait 30–60 minutes until the cold trap is sufficiently cooled or chamber pressure drops below 30 Torr.
- Ensure vacuum remains ON throughout.

11. Begin Heating Sequence

- Turn on the furnace and chamber gauge heater.
- Turn on the vaporizer heater.

12. Begin Deposition

- Press the green start button.
    - Confirm sample rotation visually through the chamber window.
- The system will:
    - Heat the furnace and gauge heaters to operating temperature.
    - Maintain vacuum until base pressure (~17 Torr) is reached.
    - Ramp up the vaporizer heater, causing dimer vaporization.
    - Pyrolyze the dimer into monomer gas, which deposits within the room temperature chamber.

- Monitor deposition:
    - Chamber pressure increases during deposition.
    - Once complete, pressure declines.
    - Upon reaching base pressure again, heaters will shut off automatically.

13. End Deposition
    - Press the green stop button.
    - Turn OFF the furnace and chamber gauge heaters.
    - Turn OFF the vaporizer heater.
    - Keep the vacuum and chiller ON.
14. Cool Down System
    - Allow the system to cool until the furnace temperature is <1000 °C (acceptable up to 1500 °C).
    - Cooling may take up to 3 hours.
    - If water condensation appears on the cold trap lid, wipe dry with a lint-free cloth.
15. Final Shutdown
    - Turn OFF the chiller.
    - Vent the chamber—this should take only a few seconds.
16. Disassemble System
    - Carefully remove the cold trap:
        - Avoid bending the tube, as cold glass is prone to cracking.
        - If water vapor condenses into ice on a room-temperature trap, allow it to melt before reuse.
    - Remove the chamber lid. This may require gentle pressure.
    - Retrieve the silicon wafer (if used).
17. Re-seal Chamber
    - Clean the chamber lid O-ring again with Scotch tape.
    - Reinstall the chamber lid.

- Set the vacuum to HOLD mode or return the chamber to atmospheric conditions as standard practice.

18. Final Steps

- Remove or save the aluminum dimer boat for reuse or disposal.
- Press the EMO button to shut down the system.
- After wafer removal, retrieve ultra-thin free-standing PaN films using tweezers.
    - Use two tweezers to prevent folding or damage due to film fragility.

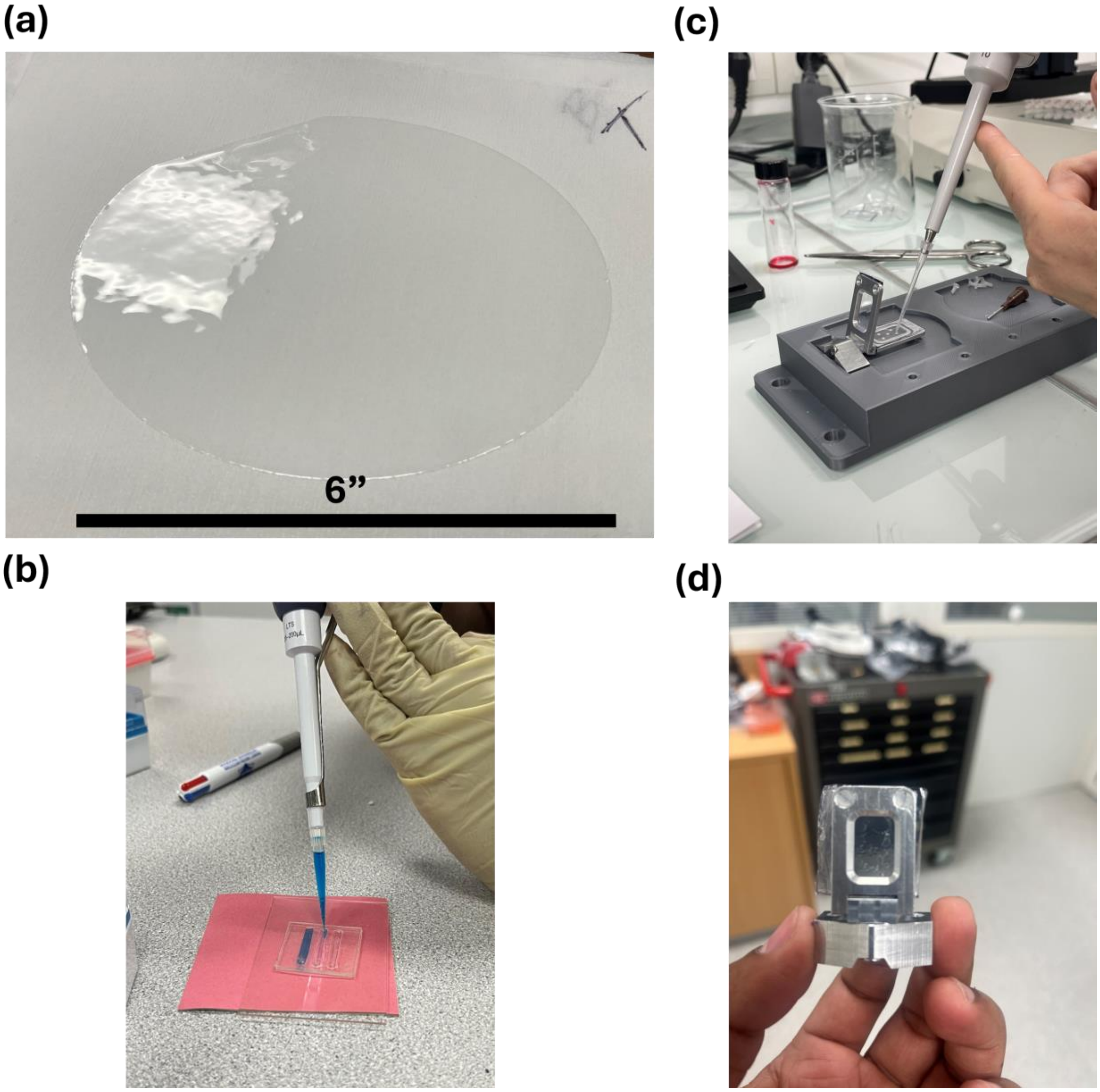

Fig. S1: a) Large area free-standing PaN film. These films are cut to size to make different types of PaN fixed-target sample platforms; b) Closed-chip PaN FT with independent windows; c) REP-24 microcrystals sample loading on film-on-film PaN FT and held in place to the actuators using SOS metal holder[49]; d) Film-on-film PaN FT loaded with REP-24 wild type protein microcrystals.

Parameters utilized for screening printing to fabricate closed chip PaN fixed-target platforms:

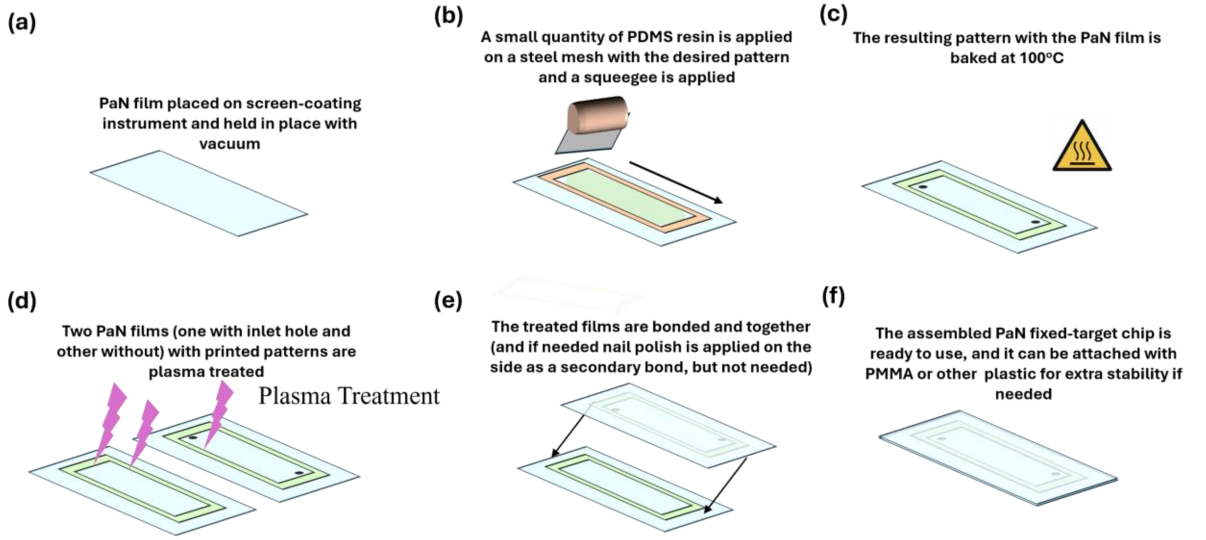

Fig. S2. Systematic Screen printing methodology of making closed-chip PaN FT. a) The PaN film is cut to size, and placed on the screen-coating instrument, and held in place with vacuum; b) A stell-mesh with the desired pattern is placed on the film, and a small quantity of PDMS resin is introduced and the squeegee is applied; c) The resulting pattern with the PaN film is baked at 100°C; d) Two patterned films are plasma treated; e) The plasma treated films are placed one-upon-another and bonded; f) The ready to use assembled devices have holes at the desired locations for crystal slurry introduction.

For screen printing, the relationship between screen parameters and ink rheological characteristics is of outmost importance. If an ink is too viscous, no matter how large the mesh opening it will not effectively pass through the mesh to achieve a successful print. However, if it is too thin, the ink will likely bleed out from the desired pattern when deposited, which is an undesirable outcome. The ideal ink is one with reasonably high viscosity and with the ability to shear-thin. This ensures they can keep their shape while in rest but also flow when exposed to large pressures like that of going through a screen. Here the ink is a PDMS mixture (10:1 ratio of base to curing agent). Mesh count (MC), which is the measure of how many threads cross each other in a square inch of screen has a large contribution to feature printability. Also depicted with its metric units, threads/cm, high mesh counts usually lead to fine feature printing when paired with the correct wire thickness and emulsion thickness. The emulsion is the coating over the mesh that masks off any areas you do not want the ink to transfer through. It also greatly influences

the quality of the print as it helps with detail resolution and ink transfer. In this work, we can see how the pairing of different MC screen openings and emulsion thicknesses led to different print thicknesses when paired with either a thick or thin ink of PDMS. Wire diameter is also an important factor in final print thickness, however, it was not varied within MCs in this study and therefore not a significant factor in this work.

MC	Viscosity	Resulting thickness	Spacer thickness	Resulting #chips
250	Thick	29	58	30
250	Thin	17.5	35	5
105	Thin	45	90	5

Screen Parameters

Screen ID	Material	Threads/cm	Wire Diameter (µm)	Mesh Opening (µm)	Emulsion Thickness (µm)
105 MC; 10 µm	Stainless Steel	41.3	76.2	178	10
105 MC; 25 µm	Stainless Steel	41.3	76.2	178	25
250 MC, 12 µm	Stainless Steel	98.4	36	66	10

Combination Mesh + Ink Viscosity on resulting thickness

	PDMS Viscosity	Screen ID	Thickness (µm)
1	Thick	105 MC; 10 µm	75
2	Thick	105 MC; 25 µm	55
3	Thick	250 MC, 12 µm	29
4	Thin	105 MC; 10 µm	48
5	Thin	105 MC; 25 µm	45
6	Thin	250 MC, 12 µm	17.5

<u>X-ray Fluence calculation:</u>

Based on the x-ray beam spot size of ~3 µm, the spot area is calculated to be 7.07 x 10^{-12} m^2.. Converting 9.6 keV photon energy in Joules, we obtain as 1.54 x 10^{-15} J. Number of photons per pulse is pulse energy/photon energy, i.e., 1.04 x 10^{12}. Photon fluence (i.e., photons per unit area) for 3 µm spot size, is 1.47 x 10^{19} photons/cm^2. Energy fluence is determined as E/A (where E is the pulse energy, and A is the spot area), which is 2.26 x 10^4 J/cm^2.

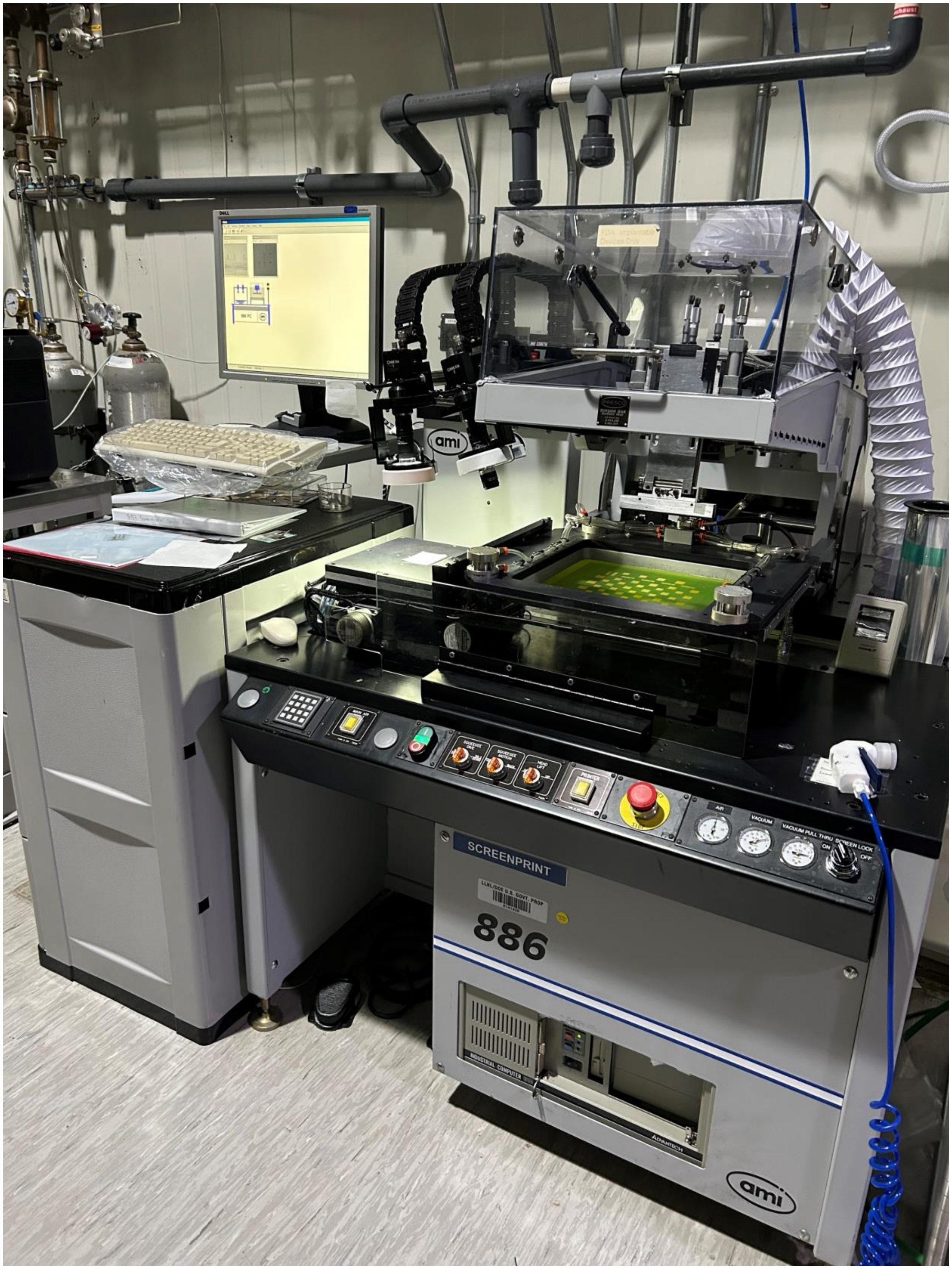

Fig S3. Automated vacuum-holder based screen printer utilized for fabricating closed chip PaN FT.
